# Elevated transgelin/TNS1 expression is a potential biomarker in human colorectal cancer

**DOI:** 10.18632/oncotarget.23275

**Published:** 2017-12-15

**Authors:** Huimin Zhou, Yiming Zhang, Lihao Wu, Wenrui Xie, Lan Li, Yu Yuan, Yu Chen, Ying Lin, Xinxiang He

**Affiliations:** ^1^ Department of Gastroenterology and Hepatology, The First Affiliated Hospital, School of Clinical Medicine of Guangdong Pharmaceutical University, Guangzhou, China; ^2^ Department of Urology, Zhujiang Hospital, Southern Medical University, Guangzhou, China; ^3^ Department of Gastroenterology and Hepatology, The Sun Yat-sen Memorial Hospital, Sun Yat-sen University, Guangzhou, China

**Keywords:** transgelin, TNS1, prognosis, colorectal cancer

## Abstract

Transgelin is an actin-binding protein that regulates cell motility and other important cellular functions. Previous studies have suggested that transgelin expression is associated with cancer development and progression, but its specific role in colorectal cancer (CRC) remains controversial. We analyzed expression of transgelin and its candidate downstream target, tensin 1 (TNS1), in CRC patients using the ONCOMINE, Protein Atlas, and OncoLnc databases. Transgelin and TNS1 mRNA and protein levels were higher in CRC patients and CRC cell lines than in normal tissues and cells. Survival analyses using the OncoLnc database revealed that elevated TAGLN/TNS1 levels were associated with a poor overall survival in CRC patients. Transgelin suppression using siRNA decreased TNS1 expression in CRC cells, demonstrating that transgelin induces the TNS1 expression. Importantly, suppression of transgelin or TNS1 using siRNA decreased proliferation and invasiveness of CRC cells. These results suggest that transgelin/TNS1 signaling promotes CRC cell proliferation and invasion, and that transgelin/TNS1 expression levels could potentially serve as a prognostic and therapeutic target in CRC patients.

## INTRODUCTION

Colorectal cancer (CRC), one of the leading causes of cancer death worldwide, is a multifaceted disease with diverse clinical, pathological, and molecular features [1]. Multiple pathways are involved in colorectal carcinogenesis and contribute to the tumor initiation and progression. Although localized CRC is curable by surgical resection, many CRC patients will experience recurrent and metastatic disease that is associated with a high morbidity and mortality [2]. Thus, identification of novel CRC biomarkers may provide valuable prognostic and therapeutic implications.

Transgelin, also known as SM22, is an actin-binding protein encoded by the *TAGLN* gene, which primarily participates in processes associated with remodeling of the actin cytoskeleton [3]. Several early studies reported that downregulation of transgelin was associated with a poor overall survival (OS) in CRC patients [4–6], suggesting that transgelin might act as a tumor suppressor. However, our recent studies have shown that transgelin promotes invasion, survival, and resistance to anoikis in CRC cells, and that transgelin upregulation is associated with metastasis in CRC patients [2, 7, 8]. Consistent with our findings, several studies from other laboratories have also suggested a similar tumor-promoting function of transgelin [9, 10]. Hence, it is important to clarify the transgelin role in CRC.

Tensin is a family of multi-domain scaffold proteins that include tensin 1, tensin 2, tensin 3, and c-ten. Tensin proteins bind to actin filaments and participate in signaling pathways [11]. Tensin 1 (TNS1) is a 220-kD protein localized to focal adhesions; it acts as a molecular bridge linking the extracellular matrix (ECM), actin cytoskeleton, and signal transduction [12, 13]. Although TNS1 is expressed in normal tissues [14], recent studies have suggested that it might be involved in tumorigenesis in several types of tumors [15–17]. Analysis of human CRC tissues using a high resolution genome-wide comparative genome hybridization has indicated that TNS1 might function as an oncogene in CRC [18]. In addition, our previous study has suggested that TNS1 might be a downstream target of transgelin. However, the specific role of TNS1 in CRC progression, and its relationship with transgelin are not clear.

In the present study, we used bioinformatics to investigate the role of transgelin and TNS1 in the OS in CRC patients, and we analyzed the TNS1 and transgelin regulation in CRC cells. Our results show that transgelin and TNS1 are enriched in CRC patients, and their expression correlates with OS. Additionally, our data demonstrate for the first time that transgelin induces TNS1 expression, leading to an increased proliferation and invasion of CRC cells. Together, our results indicate that the transgelin/TNS1 signaling axis may represent a novel therapeutic and prognostic biomarker for CRC.

## RESULTS

### Transgelin and TNS1 levels are increased in CRC patients and CRC cells

**Figure 1:**
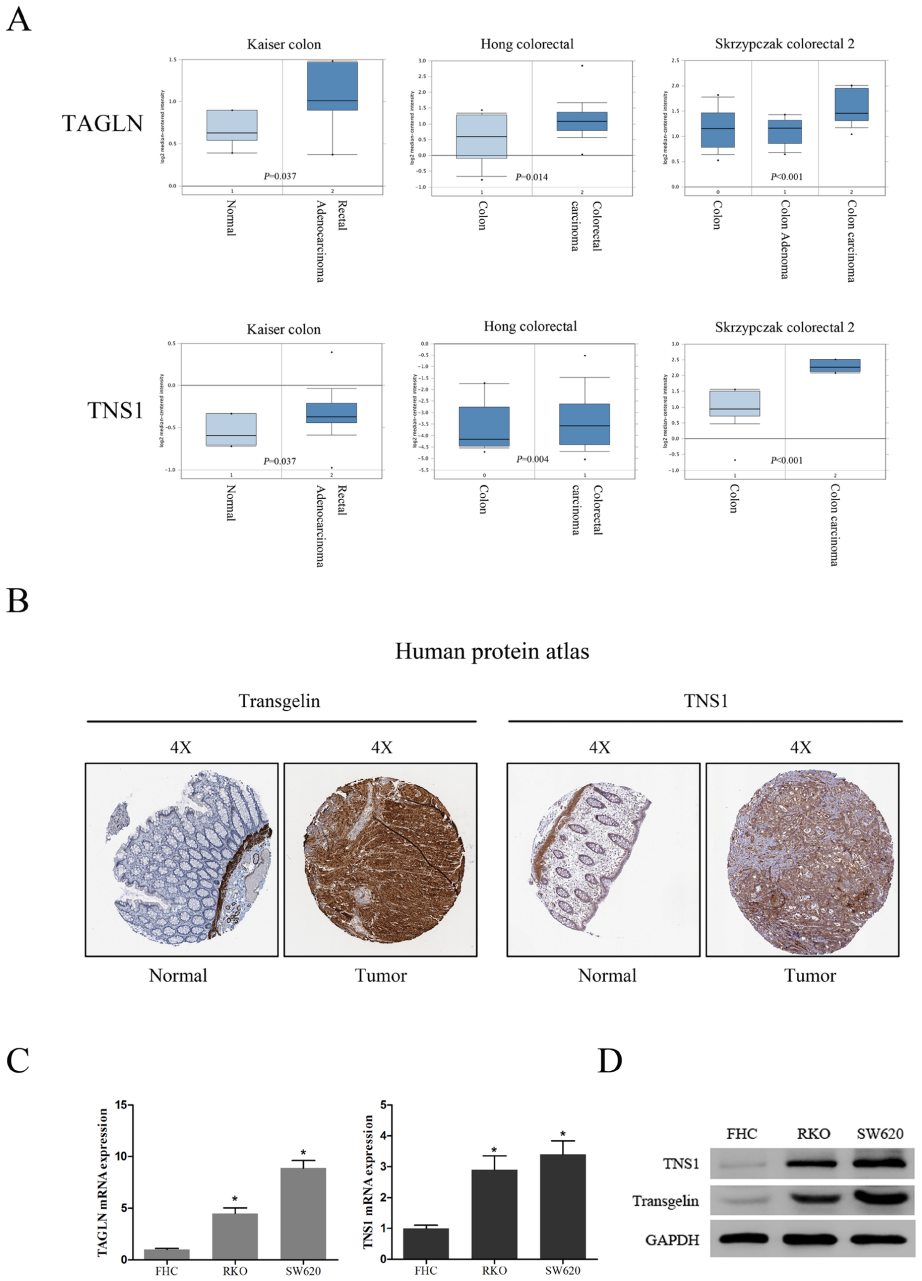
Clinical significance of transgelin and TNS1 in CRC **(A)** Oncomine data mining analysis of TAGLN and TNS1 mRNA levels in three different datasets between normal colon versus colorectal cancer. **(B)** Transgelin and TNS1 expression in normal colon tissue and colorectal specimens. Images were taken from the Human Protein Atlas (http://www.proteinatlas.org) online database. **(C)** qRT-PCR analysis of TAGLN and TNS1 showing that they are highly expressed in human CRC cell lines SW620 and RKO compared to FHC (*P* < 0.05). **(D)** Western Blot analysis showing that transgelin and TNS1 are highly expressed in SW620 and RKO cells.

To analyze transgelin and TNS1 expression in CRC patients, we first compared gene levels of *TAGLN* and *TNS1* in CRC tissues and normal samples, using the ONCOMINE database. TAGLN and TNS1 mRNA levels were significantly increased in CRC patients (*P* < 0.05; Figure 1A). Analysis of transgelin and TNS1 protein levels using the human protein atlas also revealed an increased expression of both proteins in CRC tissues, and a weak expression in normal colon samples (Figure 1B). In addition, analysis of transgelin and TNS1 levels in CRC RKO and SW620 cells demonstrated that transgelin and TNS1 mRNA and protein levels were increased in both CRC cell lines compared to normal human colon cells (FHC) (Figures 1C, 1D).

### Increased TAGLN and TNS1 mRNA levels are associated with poor OS in CRC patients

**Figure 2:**
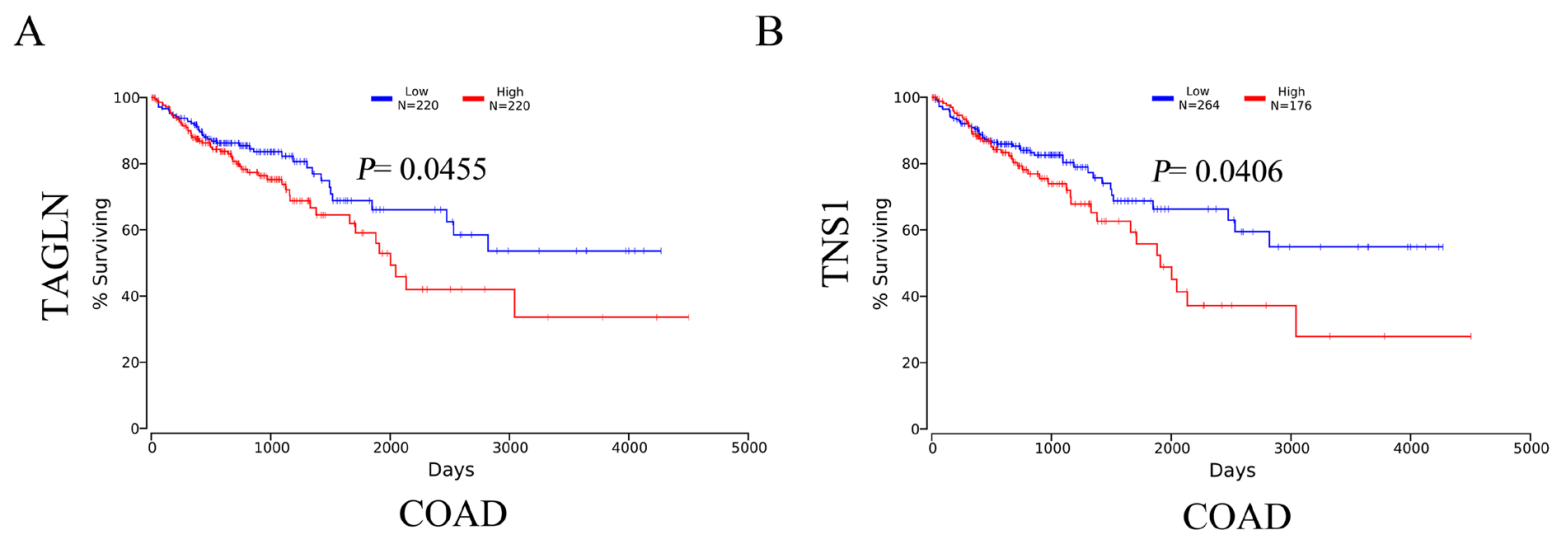
The survival analysis of mRNA levels of TAGLN **(A)** and TNS1 **(B)** in CRC patients (OS in Kaplan-Meier plotter). COAD: colon adenocarcinoma.

To investigate whether there might be a prognostic role of transgelin and TNS1 in CRC, we performed a survival analysis for TAGLN and TNS1 in CRC patients based on The Cancer Genome Atlas (TCGA) database. The Kaplan-Meier curve and log-rank test analyses revealed that TAGLN and TNS1 mRNA levels were associated with OS in CRC patients (*P* < 0.05) (Figures 2A, 2B). The CRC patients with increased mRNA levels of TAGLN and TNS1 were predicted to have worse OS.

### TNS1 is downstream target of transgelin signaling in CRC cells

**Figure 3:**
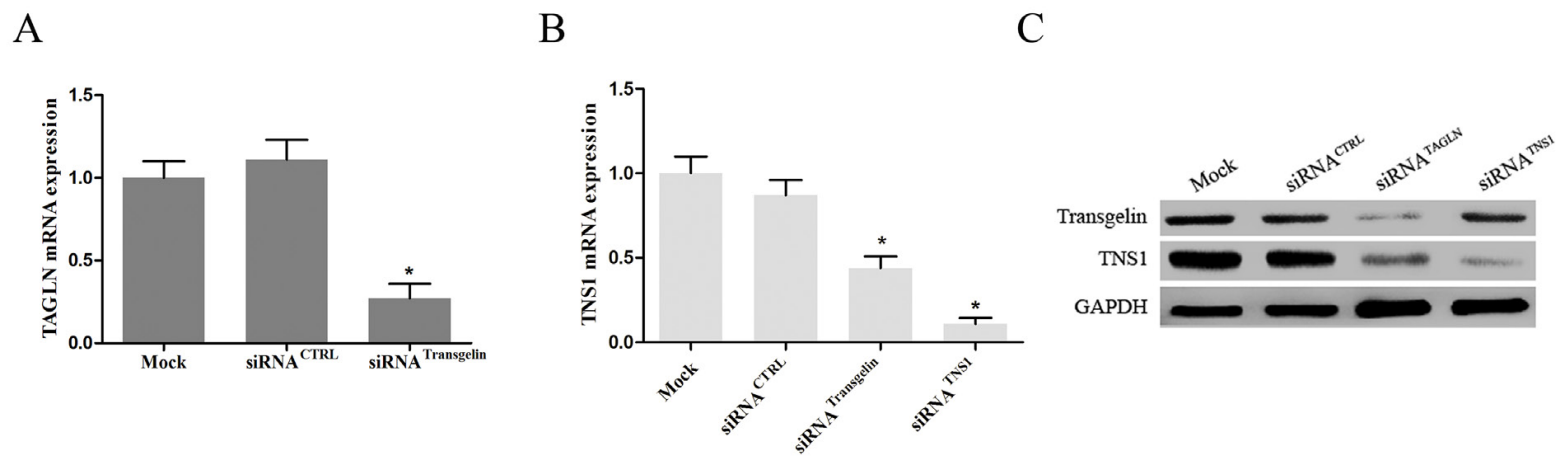
Transgelin upregulates TNS1 expression in CRC cells **(A)** qRT-PCR was performed to validate the efficiency of transgelin interference in SW620 cells (*P* < 0.05). **(B)** qRT-PCR and **(C)** Western blot analysis demonstrating that transgelin suppression reduces endogenous TNS1 mRNA and protein levels in SW620 cells (mRNA, *P* < 0.05; protein, *P* < 0.05). CTRL: control.

We have previously shown that overexpression of transgelin increases TNS1 mRNA levels in CRC cells [7]. To extend this observation, we investigated TNS1 expression in SW620 cells transiently transfected with transgelin siRNA. As shown in Figure 3A, transgelin siRNA effectively suppressed the transgelin mRNA levels (>70%) at 48 hours after transfection. Importantly, transgelin suppression markedly decreased TNS1 mRNA and protein levels (*P* < 0.05; Figure 3B), indicating that TNS1 is a downstream target of transgelin in CRC cells.

### Transgelin/TNS1 axis promotes proliferation and invasiveness of CRC cells

**Figure 4:**
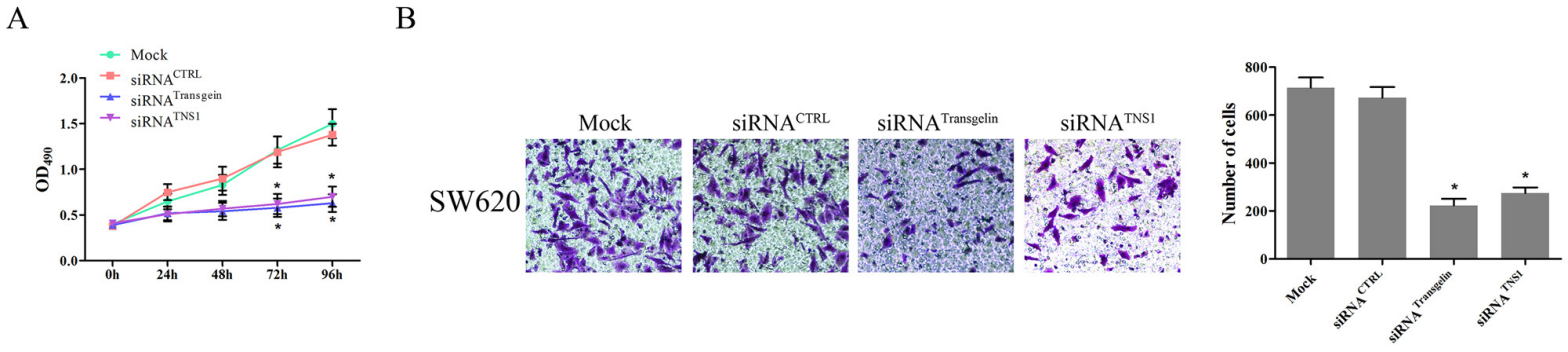
Transgelin/TNS1 axis promotes proliferation and invasiveness of CRC cells **(A)** Cell proliferation measured by MTS assay is cells transfected with control siRNA, and transgelin or TNS1 siRNA on days 3 and 4 (*P* < 0.05). **(B)** Cell invasion assay demonstrating that transgelin or TNS1 suppression decreases cell invasion compared to control siRNA (*P* < 0.05). The si-transgelin group showed results similar to those for the si-TNS1 group (*P* > 0.05).

To investigate the role of the transgelin/TNS1 axis in the regulation of CRC cell proliferation, we analyzed proliferation of SW620 cells transfected with control and transgelin or TNS1 siRNA using the MTS assay. As shown in Figure 4A, compared to cells transfected with control siRNA, transfection with transgelin or TNS1 siRNA suppressed the proliferation capacity of SW620 cells at 72 and 96 hours (*P* < 0.05), suggesting that the transgelin/TNS1 signaling axis induces proliferation of CRC cells.

Since cell invasiveness is closely related to cancer metastasis, we further evaluated the metastatic properties of SW620 cells using the cell invasion assay. As shown in Figure 4B, both transgelin and TNS1 siRNA significantly inhibited the invasion capability of SW620 cells compared to control siRNA (*P* < 0.05), indicating that the transgelin/TNS1 axis promotes invasiveness of CRC cells.

## DISCUSSION

In recent years, colorectal cancer has been the leading cause of cancer-related death in China [21, 22]. Surgery, radiotherapy, and chemotherapy used alone or in combination, are commonly employed for the treatment of CRC patients. Molecular targeted therapies are a promising novel approach in CRC, especially in metastatic CRC. As an abundant protein of smooth muscle cells and an important factor regulating the actin cytoskeleton dynamics, transgelin has been reported to be involved in many cancer-related processes [3]. Our previous studies have suggested that transgelin may be an oncogenic protein that participates in the CRC lymphatic metastasis [2, 7]. However, the specific transgelin role in CRC is still contradictory.

TNS1, also known as Tensin 1, has actin cross-linking capacity and binds to actin filaments when participating in signaling pathways. TNS1 is involved in dis/assembly of focal adhesions [23], integrin translocation [24], maintenance of normal renal function [25], and wound healing [26]. More importantly, prior studies have reported that increased expression of TNS1 correlates with tumor cell migration and identified TNS1 as a candidate oncogene in CRC [13, 18]. However, the mechanisms regulating the TNS1 expression are not known. Using microarray analysis, our previous studies have indicated that overexpression of transgelin induces TNS1 expression in CRC cells, but the specific mechanisms are not fully understood.

In this study, we demonstrate that the expression of both transgelin and TNS1 is increased in CRC tissues and cells. Moreover, the survival analysis revealed that high expression of transgelin and TNS1 correlates with poor survival in CRC. Our results demonstrate that transgelin suppression inhibits the TNS1 expression in SW620 cells, indicating that transgelin induces TNS1 expression in CRC cells. In addition, our results show that the transgelin/TNS1 axis promotes proliferation and invasiveness of CRC cells. To our knowledge, these data are the first to indicate the prognostic role of transgelin and TNS1 in CRC, and to demonstrate that transgelin induces TNS1 expression.

In conclusion, our data demonstrate that the transgelin/TNS1 axis is activated in colorectal cancer, suggesting that it may serve as a potential therapeutic and prognostic biomarker for CRC.

## MATERIALS AND METHODS

### Cell culture

SW620 cells were obtained from ATCC and maintained in RPMI-1640 medium supplemented with 10% fetal bovine serum (FBS) (Gibco, USA). RKO cells were cultured in MEM medium supplemented with 10% FBS. FHC cells were purchased from ATCC and cultured in DMEM/F12 medium supplemented with 10% FBS (Gibco, USA), 10 ng/ml cholera toxin (Sigma, USA), 0.005 mg/ml insulin (Roche, USA), 0.005 mg/ml transferrin (Sigma, USA), and 100 ng/ml hydrocortisone (Sigma, USA). Cells were grown in a humidified incubator at 37°C with 5 % CO_2_.

### Bioinformatics analysis of transgelin and TNS1 expression and OS in human CRC

The protein levels of transgelin and TNS1in CRC tissues and normal tissues were determined from the human protein atlas (www.proteinatlas.org). Both transgelin and TNS1 gene expression in CRC specimens and normal tissues is available through *Oncomine* (Compendia Biosciences, www.oncomine.org) [19]; the cut-off *p* value and fold change were defined as 0.01 and 2, respectively. Survival analyses were performed using *OncoLnc* (www.oncolnc.org) [20].

### Western blotting

Cells were lysed in RIPA buffer (Beyotime, China) containing protease inhibitors (complete Mini, EDTA-free Protease Inhibitor Cocktail, Roche, USA). Protein lysates were centrifuged, and the supernatants were collected for protein quantification using the bicinchoninic acid assay kit (Pierce, USA). Protein lysates were separated through 8–10% SDS-PAGE (Beyotime, China) and transferred to nitrocellulose membranes (Millipore, USA). Membranes were then probed with antibodies against transgelin (1:1000, ab155272 Abcam, USA) and TNS1 (1:1000, ab167660, Abcam, USA). GAPDH (1:800, cat. No. 5174, Cell Signaling Technology, USA) was used as a protein loading control. Following an incubation with HRP-conjugated goat anti-rabbit or anti-mouse IgGs (1:1000, Cell Signaling Technology, USA), the proteins were visualized with Syngene G:BOX Chemi XT4 fluorescence and chemiluminescence gel imaging system (Cambridge, UK).

### RNA extraction, reverse transcription, and quantitative real-time PCR (qRT-PCR)

Total RNA was extracted using TRIzol reagent (Invitrogen Life Technologies, USA) and reverse transcribed with the RT kit from TaKaRa Biotechnology Co. Ltd. (Dalian, China). The primers used were as follows: for transgelin, forward: GTTCCAGACTGTTGACCTCTTT, reverse: CTGCGCTTTCTTCATAAACC; for TNS1, forward: GTACGTCACAGAGAGGATCATCG, reverse: GCAGGTAGTTGCCTCCATGTT; for GAPDH (endogenous control), forward: TGGTGAAGACGCCAGTGGA, reverse: GCACCGTAAGGCTGAGAAC. The qRT-PCR was performed using a Light Cycler® 480 SYBR Green I Master mix (Roche, USA) on a Light Cycler® 480 System (Roche, USA) according to the manufacturer’s instructions. The PCR conditions were as follows: 95°C for 30s, 35 cycles at 95°C for 5s, then 60°C for 30s. The relative mRNA expression was calculated using the 2^-∆∆Ct^ comparative CT method.

### Small interfering RNAs (siRNAs) transfection

Effective siRNA targeting of human transgelin was performed as described previously [2]; TNS1 siRNA was purchased from Sigma-Aldrich. SW620 cells (1×10^6^) were seeded in six-well plates and transfected using RNAiMAX reagent (Invitrogen Life Technologies, USA) for 48h. Subsequently, qRT-PCR analysis was performed to verify the suppression of transgelin and TNS1 mRNA expression.

### Cell proliferation assay

For cell proliferation assay, four groups of SW620 cells were seeded in 96-well plates at 2.0×10^3^cells/well in a final volume of 100μL and incubated overnight. The cell viability was determined with CellTiter 96 non-radioactive cell proliferation assay (MTS) (Promega BioSciences, USA) following the manufacturer's protocol. All experiments were performed in triplicates.

### Cell invasion assay

The invasion assay was performed using the Bio-Coat Matrigel invasion assay system (BD Bioscience, USA) following the manufacturer’s protocol. Four groups of SW620 cells were suspended in serum-free RPMI-1640 medium and seeded into the upper chambers of 24-well trans-well plates. FBS (10 %) was added to the bottom chambers. After 24 h, the cells on the upper side were removed with a cotton swab, while the cells on the bottom side of the filter were fixed with 4% paraformaldehyde, stained with crystal violet, and counted. The invasive rate was expressed as a percentage of control. All experiments were performed in triplicates.

### Statistical analysis

Statistical analyses were performed using SPSS 20.0 software (SPSS Inc., USA). The values were expressed as mean ± standard deviation (SD) of three independent experiments, and the significance of the differences among three or four groups was calculated using one-way analysis of variance (ANOVA) while Student's *t*-test was used to ascertain the significance between two groups. *P* values < 0.05 were considered to be statistically significant.

## Abbreviations

CRC: colorectal cancer
OS: overall survival
qRT-PCR: quantitative real-time PCR
siRNA: small interfering RNAs
SD: standard deviation
